# An Experience as a Medical Support during the COVID-19 Pandemic

**DOI:** 10.31729/jnma.7554

**Published:** 2022-07-31

**Authors:** Sachi Adhikari, Saloni Shrestha

**Affiliations:** 1 Nepaigunj Medical College and Teaching Hospital, Kohalpur, Banke, Nepal; 2 Nepal Medical College and Teaching Hospital, Attarkhel, Kathmandu, Nepal

**Keywords:** *COVID-19 pandemic*, *medical students*, *telehealth*, *volunteerism*

## Abstract

In the midst of the COVID-19 pandemic, volunteering enabled us to support people facing difficulties in locating healthcare facilities. Working as a medical support volunteer allowed us to interact closely with patients. We assisted them by providing information on the availability of hospital beds, intensive care unit beds, ventilators and oxygen cylinders. We had numerous beautiful as well as dreadful experiences and learned valuable lessons during the process. We experienced a wide range of emotions, from the joy of informing about the availability of a vacant bed to the guilt and dilemma of having to prioritise in such a crisis. We learnt about how an empathetic approach and active listening helps to connect people.

## INTRODUCTION

Coronavirus Disease-19 (COVID-19) is an infectious disease of the respiratory system caused by SARS-COV-2.^1^ In May 2021, the second wave of COVID-19 plunged Nepal into a public health catastrophe.^2^ As a developing country, it lacked the resources to deal with the soaring cases. Nepal's healthcare facilities were limited to 26,930 hospital beds, 1595 Intensive Care Unit (ICU) beds and 840 ventilators, out of which only one-third were assumed to be used for COVID-19 cases.^3^ Due to overcrowding in COVID units and ICUs, several people died from a lack of oxygen cylinders, ICU beds and ventilators. In the midst of a healthcare crisis, COVID Connect Nepal, a volunteer-run organisation assisted people to locate hospital beds, ICU beds, oxygen cylinders and ventilators after the verification of live requests on the COVID Connect Nepal website.^4^

## VOLUNTEERING JOB AS A MEDICAL SUPPORT

Amidst the havoc caused by the COVID-19 pandemic, we as medical students were more than eager to help manage the catastrophe. We were motivated to volunteer in COVID Connect Nepal with the hopes of alleviating people's suffering, be it by aiding people to locate available healthcare facilities or at least consoling them in such difficult times. Our medical team initially conducted an online conference where a duty plan was formulated and telemedicine guidelines and telephone triage was discussed. Telemedicine is a way of providing medical care through interactive telecommunications. Telecommunication can be done via various technologies, one of them being telephones.^5^ Telephone triage is a telephone-based service which is used to assess and manage healthcare facilities based on urgency. Telephone triage can be used to provide health information, signposting to other services, direct referrals and give advice.^6^

We were assigned as medical support and were required to work in shifts as COVID Connect Nepal offered a twenty-four-hour telemedicine service.^4^ Our shifts were assigned to us based on our availability. We were given an id to log in to COVID Connect Nepal's website and assess and process live requests for hospital beds, ICU beds, oxygen cylinders, and ventilators. We contacted the requestors, listened to their concerns, acquired a brief history of the patients and categorised urgency based on their health status such as oxygen saturation level, temperature, and co-existing medical conditions. As medical support, we used to counsel and console frightened patient parties before forwarding the categorised list to the data team who would then inquire about hospitals near the patient's area. In response to their requests, we would advise patients about vacant beds, oxygen cylinders, and ventilators if available.

## EXPERIENCE AS A MEDICAL SUPPORT

In a span of a few months, we travelled through a roller coaster of emotions. We were eager to inform the patients about the availability of hospital beds in the hopes of saving their lives. Regardless, we were heartbroken to learn that a vacant bed sometimes meant the demise of a patient admitted there previously. One moment we felt helpless scanning through hundreds of live requests although the other moment we felt accomplished after receiving a “thank you" message from a patient's relatives. Periodically, a wave of shame would wash over us when we were alerted that there was just one vacant bed available. Who should we contact first? For every person on the request list, we wished to alleviate the pain of their beloved ones. Worst of all was a follow-up phone call with the patient's dear ones only to find out that the patient who was alive a day before, died. To deal with this mental toll, we could have practised meditation and sought additional help. Throughout the journey, we faced many challenges. One of them was being infected with COVID-19 itself. Having worked for a while, the worsening pandemic didn't let us allow ourselves to rest. To cope with this physical and mental toll, COVID Connect Nepal used to organise refreshment programs where volunteers use to sing, recite poems and share their respective experiences. Despite the ups and downs, COVID Connect Nepal was more than simply a volunteer opportunity, it was an emotional event that brought us closer to the patients and the health care system. As medical support, we felt a sense of purpose we hold for our community and realised that an empathetic approach is a key element in volunteering.

## LEARNING FROM VOLUNTEERING

As fourth-year medical students, clinical rotations were introduced to us not long ago. Working in COVID Connect Nepal gave us an opportunity to dive deeper into our subject and have a better understanding of what medicine really was. It wasn't just an art of applying medical knowledge; it was also an art of communication. In a telemedicine setting, we learned to interact effectively while obtaining patients' histories. We practised active listening, information gathering and empathising. We also learned about collaboration, and teamwork. We firmly believe that the skills we acquired throughout the journey will further improve our efficiency in health care and lead to greater patient satisfaction. Every individual possesses unique skills. When all of these varied skills were brought together and pooled for a single aim, that was when COVID Connect Nepal was able to effectively tackle the issue. Along with the good aspect, we also realised that we sometimes tend to forget to take care of ourselves and our mental health while being highly driven to take care of our fellow beings. To conclude, we were delighted to discover a platform that provided us with a sense of purpose in our spare time. It was used wisely by assisting those in need and forging new connections.

## WAY FORWARD

Volunteering is an act of kindness, a sense of responsibility towards our community and moreover volunteering helps to reflect on ourselves as a person. Volunteering aids in developing various skills such as teamwork, communication skills, and an empathetic approach to connecting with people which are key elements of healthcare. Working as medical support in a telemedicine setting forges the practice of active listening and information gathering. It is a new experience in itself to build new connections without face-to-face interaction. We, as medical students shouldn't only confine ourselves to books but also we need to exercise the very purpose of medicine, which is making a difference in someone's life and alleviating one's suffering. One can do so through active volunteering.
